# Increasing Trends of Association of 16S rRNA Methylases and Carbapenemases in *Enterobacterales* Clinical Isolates from Switzerland, 2017–2020

**DOI:** 10.3390/microorganisms10030615

**Published:** 2022-03-14

**Authors:** Claudine Fournier, Laurent Poirel, Sarah Despont, Julie Kessler, Patrice Nordmann

**Affiliations:** 1 Medical and Molecular Microbiology Unit, Faculty of Science and Medicine, University of Fribourg, 1700 Fribourg, Switzerland; claudine.fournier@unifr.ch (C.F.); sarah.despont@students.unibe.ch (S.D.); 2 INSERM European Unit (IAME, France), University of Fribourg, 1700 Fribourg, Switzerland; 3 Swiss National Reference Center for Emerging Antibiotic Resistance (NARA), 1700 Fribourg, Switzerland; julie.kessler@unifr.ch; 4 Institute for Microbiology, University of Lausanne and University Hospital Centre, 1011 Lausanne, Switzerland

**Keywords:** carbapenemases, 16S rRNA methylases, *Enterobacterales*, association, co-occurrence

## Abstract

Aminoglycosides (AGs) in combination with β-lactams play an important role in antimicrobial therapy in severe infections. Pan-resistance to clinically relevant AGs increasingly arises from the production of 16S rRNA methylases (RMTases) that are mostly encoded by plasmids in Gram-negative bacteria. The recent emergence and spread of isolates encoding RMTases is worrisome, considering that they often co-produce extended-spectrum β-lactamases (ESBLs) or carbapenemases. Our study aimed to retrospectively analyze and characterize the association of carbapenem- and aminoglycoside-resistant clinical isolates in Switzerland during a 3.5-year period between January 2017 and June 2020. A total of 103 pan-aminoglycoside- and carbapenem-resistant clinical isolates were recovered at the NARA (Swiss National Reference Center for Emerging Antibiotic Resistance) during the 2017–2020 period. Carbapenemase and RMTase determinants were identified by PCR and sequencing. The characterization of plasmids bearing resistance determinants was performed by a mating-out assay followed by PCR-based replicon typing (PBRT). Clonality of the isolates was investigated by multilocus sequence typing (MLST). Over the 991 *Enterobacterales* collected at the NARA during this period, 103 (10.4%) of them were resistant to both carbapenems and all aminoglycosides. Among these 103 isolates, 35 isolates produced NDM-like carbapenemases, followed by OXA-48-like (*n* = 23), KPC-like (*n* = 21), or no carbapenemase (*n* = 13), OXA-48-like and NDM-like co-production (*n* = 7), and VIM-like enzymes (*n* = 4). The RMTases ArmA, RmtB, RmtC, RmtF, RmtG, and RmtB + RmtF were identified among 51.4%, 13.6%, 4.9%, 24.3%, 1%, and 1%, respectively. Plasmid co-localization of the carbapenemase and the RMTase encoding genes was found among ca. 20% of the isolates. A high diversity was identified in terms of the nature of associations between RMTase and carbapenemase-encoding genes, of incompatibility groups of the corresponding plasmids, and of strain genetic backgrounds, highlighting heterogeneous importations rather than clonal dissemination.

## 1. Introduction

The increasing occurrence of resistance to last-resort antimicrobials, including carbapenems and aminoglycosides, in clinically relevant Gram-negatives, including *Enterobacterales*, is currently of great concern. Aminoglycosides play an important role in antimicrobial therapy in severe infections, usually given in combination with β-lactam agents. Aminoglycoside-modifying enzymes, namely phosphotransferases, adenylyltransferases, nucleotidyltransferases, and acetyltransferases, have been identified for decades as a common source of acquired resistance to aminoglycoside molecules. Each of those enzymes may confer resistance to some but not all of the commonly used molecules, namely amikacin, gentamicin, tobramycin, and kanamycin, although the combination of those enzymes in single strains is possible. Lately, 16S rRNA methylases (RMTases) have been identified as a source of acquired resistance to aminoglycosides. All of them methylate the target of aminoglycosides, namely the 16S rRNA, and consequently confer high-level and broad-spectrum resistance to all clinically relevant aminoglycosides. They are mostly encoded by plasmids and found among many different Gram-negative bacteria. To date, ten RMTases have been identified (namely ArmA, RmtA-H, and NpmA, including a series of variants within these groups) [1]. The recent emergence and spread of those genes encoding RMTases is of significant concern as they are often acquired by enterobacterial isolates that produce extended-spectrum β-lactamases (ESBLs) or carbapenemases, and more specifically isolates producing metallo-ß-lactamases of the NDM type. Noticeably, among the 10 RMTases described so far, the most widespread enzymes are ArmA and RmtB.

Carbapenems are considered last-resort antibiotics, due of their broad spectrum of activity, but their efficacy can be compromised by the production of inactivating enzymes, namely carbapenem-hydrolyzing ß-lactamases or carbapenemases. Carbapenemases encountered among *Enterobacterales* are categorized in three different biochemical Ambler classes: class A, including KPC, GES, and IMI serine β-lactamases; class B, including NDM, VIM, and IMP metallo-β-lactamases; and class D, including OXA-48-like β-lactamases [2].

With the emergence of carbapenemase-producing Gram-negative bacteria, polymyxins, including colistin, have gained a renewed interest as a last option in treatment of severe infections caused by MDR. However, the use of polymyxins has been associated with an increased detection polymyxin-resistant isolates, which is particularly worrying, because it may lead to impossible-to-treat infections [3,4].

It was shown during the last decade that the occurrence of RMTases was particularly frequent among NDM-producing *Enterobacterales*, which was either due to the acquisition of distinct plasmids encoding a carbapenemase and an RMTases by given isolates, or (though to a lower extent) to the acquisition of plasmids carrying both RMTase and NDM encoding genes [5]. Recent reports from different geographical areas showed that the co-production of RMTases and carbapenemases was on the rise in *Enterobacterales*, such as in Spain with RmtF dominating over other RMTases and mainly being identified in *Klebsiella pneumoniae* and *Enterobacter cloacae* bearing the *bla*_OXA-48_, *bla*_NDM-1_*,* and *bla*_VIM-1_ carbapenemase genes, or in Greece with RmtB and KPC being the dominating RMTase and carbapenemase enzymes, respectively [6,7]. Similarly, a study performed in China showed a high prevalence of RMTases among carbapenem-resistant *K. pneumoniae* clinical isolates associated with bloodstream infections in eleven hospitals [8]. The dominant RMTase in that collection was RmtB, while the most common carbapenemases were of KPC type. A single clone corresponding to a KPC-producing ST11 was shown to be predominant and widely disseminated (seven provinces).

Owing the increasing reports of association between carbapenemases and RMTases worldwide, we aimed to evaluate this worrying phenomenon among clinical enterobacterial isolates in Switzerland recovered through our National Reference Center for Antibiotic Resistance. Thus far, only a few reports of RMTase-producing *Enterobacterales* from Switzerland are known [9]. Several reports corresponded to importations from abroad, such as a KPC-2 + RmtG-positive *K. pneumoniae* from Brazil, or an NDM-1 + OXA-181 + ArmA *K. pneumoniae* from Serbia, but some autochthonous cases were noticed, including an ST231 *K. pneumoniae* co-producing OXA-232 and RmtF identified in different parts of the country [10,11,12].

## 2. Materials and Methods

### 2.1. Bacterial Isolates

Pan-aminoglycoside (amikacin, tobramycin, gentamicin, and kanamycin)-resistant and carbapenem-resistant enterobacterial isolates collected at the NARA (Swiss National Reference Center for Emerging Antibiotic Resistance) during a 3.5-year period between January 2017 and June 2020 were included in the study, over a total 991 enterobacterial isolates received. A total number of 103 isolates from 80 different patients were recovered. Those strains had been isolated from wound, expectoration, blood, urine, skin, tracheal, or rectal specimens in different wards of 18 hospitals, 11 private laboratories, and one rehabilitation center, corresponding to locations scattered throughout the whole country.

Each isolate was cultured overnight on an Uri4 select (BioRad, Crissier, Switzerland) plate. Identification of the isolates at the species level was performed using the API20E system (bioMérieux, La Balme-les-Grottes, France) and submitted to the Carba NP test to assess the potential carbapenemase activity [13].

### 2.2. Susceptibility Testing

An antimicrobial susceptibility antibiogram was performed using a disk diffusion assay and interpreted according to EUCAST recommendations [14]. Susceptibility to polymyxins was first evaluated by using the Rapid Polymyxin NP test [15]. Then, the broth microdilution method was used to precisely determine the MICs of colistin of those isolates being positive with the Rapid Polymyxin NP test, namely the colistin-resistant isolates, with the MIC results being interpreted according to EUCAST 2021 guidelines [14].

### 2.3. Molecular and Immunochromatographic Analyses

The NG Carba5 immunochromatography test (NG Biotech, Guipry-Messac, France) followed by a PCR with specific primers were conducted to identify the carbapenemase content of all isolates as previously reported [16]. In order to identify the exact nature of the carbapenemase variant, amplicons were sequenced using the Sanger method by a Microsynth (Balgach, Switzerland). The carbapenem-resistant but carbapenemase-negative isolates were screened for other mechanisms of resistance, including the production of ESBL, AmpCs, efflux pumps, and impermeability [17,18].

According to the antibiogram phenotype, isolates resistant to amikacin, gentamicin, kanamycin, and tobramycin were tested by using the Rapid Aminoglycoside NP test, and screened further for 16S RMTases including ArmA, NpmA, and RmtA to RmtH by PCR with specific primers as previously described [19,20].

Colistin-resistant isolates were screened by PCR for *mcr-1* to *mcr-9* genes content [21,22]. Chromosomal genes known to be possibly involved in acquired resistance to polymyxins were PCR-amplified, and amplicons were further sequenced. Clonality of the *K. pneumoniae* and *E. coli* isolates was evaluated by multilocus sequence typing (MLST) (Pasteur scheme), while that of the *Enterobacter xiangfangensis* and *Enterobacter hormaechei* isolates was performed based on whole genome sequencing data [23,24].

### 2.4. Plasmid Characterization

Characterization of the plasmids bearing the 16S RMTase and carbapenemase genes, as well as the putative co-localization of those different genes, was evaluated by mating-out assays using azide-resistant *E. coli* J53 as the recipient strain. Transconjugants were selected on Luria-Bertani (LB) agar plates supplemented with sodium azide 100 µg/mL, gentamicin 50 µg/mL and amikacin 50 µg/mL for methylase producers, sodium azide 100 µg/mL and ceftazidime 30 µg/mL for VIM- and NDM-producers, sodium azide 100 µg/mL and temocillin 1 µg/mL for OXA-48-like-producers, and sodium azide 100 µg/mL and cefoxitin 1 µg/mL for KPC-producers. Plasmid extraction using the Zymo PURE kit (Zymo, Irvine, CA, USA) followed by electro-transformation of TOP10 recipient *E. coli* were performed for some KPC-positive isolates when mating-out assays remained unsuccessful. Selection was made on an LB agar plate supplemented with 100 mg/L of ticarcillin. The success of the transformation or conjugation weas confirmed by PCR with the corresponding 16S RNA methylase and carbapenemase-specific primers on transformants and transconjugants. PCR-based replicon typing (PBRT) was used to identify the plasmid incompatibility group [25].

## 3. Results

### 3.1. 16S rRNA Methylase and β-Lactamase Content of the Isolates

Among the 103 aminoglycoside- and carbapenem-resistant enterobacterial isolates, 99 were RMTases producers and 90 were carbapenemase producers (see Table 1 and Table 2).

Over the 99 RMTases producers, 53 isolates (53%) harbored the *armA* gene (34 *K. pneumoniae*, six *P. mirabilis*, five *Providencia* spp., four *E. coli*, four *Enterobacter* spp.), 25 (2.5%) harbored the *rmtF* gene (25 *K. pneumoniae*), 14 (1.4%) harbored the *rmtB* gene (seven *K. pneumoniae* and seven *E. coli*), five (0.5%) harbored *rmtC* (two *Enterobacter* spp., one *E. coli*, one *M. morganii*, and one *C. freundii*), one (0.1%) *K. pneumoniae* harbored the *rmtG* gene, and one (0.1%) *K. pneumoniae* co-harbored *rmtB* and *rmtF* genes.

Among the 90 carbapenemase-positive isolates, 42 isolates produced NDM-like enzymes. The *bla*_NDM-1_ and *bla*_NDM-5_ genes were identified among 30 and 12 isolates, respectively, including *K. pneumoniae* (*n* = 20), *E. coli* (*n* = 10), *Enterobacter* spp. (*n* = 6), *Morganellaceae* (*n* = 5), and *C. freundii* (*n* = 1). Seven of them co-harbored a *bla*_OXA-48-like_ gene, namely *bla*_OXA-181_ (*n* = 3), *bla*_OXA-48_ (*n* = 3), and *bla*_OXA-232_ (*n* = 1).

A total of 21 isolates were positive for KPC-like encoding genes (16 *bla*_KPC-2_ and 5 *bla*_KPC-3_), all being *K. pneumoniae*.

In addition, 23 isolates were positive for OXA-48-like encoding genes (disregarding those co-producing multiple carbapenemases as listed above), with twenty being *K. pneumoniae,* two *P. stuartii,* and a single *E. coli*. The *bla*_OXA-48_ gene was identified in eleven *K. pneumoniae* and two *P. stuartii*, the *bla*_OXA-232_ gene in six *K. pneumoniae*, and the *bla*_OXA-181_ in three *K. pneumoniae* and one *E. coli*.

Finally, four isolates were positive for VIM-like encoding genes, all being *P. mirabilis* (VIM-1, *n* = 3 and VIM-2, *n* = 1).

### 3.2. Antimicrobial Resistance Features among Colistin-Resistance Isolates

Rapid Polymyxin NP gave positive results for nine isolates (disregarding species being intrinsically resistant to colistin, i.e., *Proteus* spp.), all being *K. pneumoniae.* Susceptibility testing showed MICs of colistin at 8 µg/mL (*n* = 2), 16 µg/mL (*n* = 3), 32 µg/mL (*n* = 3), and 64 µg/mL (*n* = 1). All isolates were negative for the *mcr-1* to *mcr-9* genes. Mutations in the PmrB sequence (A246T (*n* = 5)) and T157P (*n* = 1)) were identified in six isolates, in the MgrB sequence (deletion of start codon) in two isolates, and in the PmrB (A246T) and MgrB sequences (insertion sequence IS*5*-like inserted at amino acid position 25) in a single isolate. All those mutations were identified in chromosomally encoded proteins known to be involved in acquired resistance to colistin [15].

The nine colistin-resistant *K. pneumoniae* isolates co-harbored OXA-48 and ArmA (*n* = 4), KPC-like and ArmA (*n* = 2), OXA-48, NDM-1, and RmtC (*n* = 1), and two pan-aminoglycoside-resistant KPC-producing isolates without RMTases.

### 3.3. Species and Clonal Evaluations

Overall, *K. pneumoniae* was the most common species producing RMTases with 72 isolates of 15 different STs including ST231 (*n* = 21), ST101 (*n* = 12), ST147 (*n* = 9), ST16 (*n* = 8), ST11 (*n* = 6), 437 (*n* = 4), ST944 (*n* = 2), ST395 (*n* = 3), ST14 (*n* = 1), ST15 (*n* = 1), ST35 (*n* = 1), ST247 (*n* = 1), ST340 (*n* = 1), ST405 (*n* = 1), and ST1519 (*n* = 1). *E. coli* was the second most common species with 12 isolates belonging to 8 different STs including ST354 (*n* = 3), ST167 (*n* = 2), ST8714 (*n* = 2), ST2520 (*n* = 1), ST90 (*n* = 1), ST405 (*n* = 1), ST2851 (*n* = 1), and ST6823 (*n* = 1). See Table 3 and Table 4 for details.

### 3.4. Origin of the Isolates

The majority of isolates had been recovered from rectal screening swabs and urine (respectively, *n* = 45 and *n* = 23), 14 isolates had been recovered from either throat-nose-inguinal-axis screening swabs, 6 from tissue biopsies, 6 from respiratory tracts, 4 from wounds, 2 from blood cultures, and one from an abdominal catheter, with clinical data lacking for two isolates.

### 3.5. Association between the RMTase ArmA and Carbapenemase

Among the 103 selected isolates, 53 produced ArmA, therefore being the most identified (51.4%) RMTase among carbapenem- and pan-aminoglycoside-resistant isolates. Over the 53 ArmA-producing isolates, 23 isolates co-produced NDM-like enzymes. A majority of those isolates carried the two corresponding genes (*armA* and *bla*_NDM_) onto IncA/C-type plasmids (*n* = 18) thus explained the co-occurrence [26]. For two isolates, the *bla*_NDM-1_ gene was carried by an IncA/C-type plasmid, while the *armA* gene was identified on an IncR-type plasmid. Another two isolates carried *bla*_NDM-1_ and *armA* genes on IncL/M- and IncFII-type plasmids, respectively [27,28].

Nine *armA*-producing *K. pneumoniae* were positive for *bla*_KPC_, that latter gene being carried by an IncFII-type plasmid. Five out of these nine isolates co-harbored the two genes on a single plasmid, corresponding to the widely distributed pKpQIL-IT plasmid [29,30]. In the four other isolates, *armA* was carried on a different plasmid scaffold, corresponding either to IncX3, IncL/M, IncA/C, or IncR plasmids. All these ArmA + KPC isolates belonged to ST101, with the exception of a single ST16. 

Ten *armA*-positive *K. pneumoniae* isolates possessed a *bla*_OXA-48_ gene, with the RMTase always being identified on a distinct plasmid. The *bla*_OXA-48_ gene was either located on the widespread IncL plasmid pOXA-48a (*n* = 5) or on IncFIB-type (*n* = 3), IncA/C-type (*n* = 1), or PBRT non-typeable (*n* = 1). The *armA* gene was mostly carried by an IncA/C-type plasmid (*n* = 9), except for a single isolate carrying an *armA*-positive IncR plasmid (*n* = 1). Two isolates, namely a single *K. pneumoniae* and a single *E. coli* isolate, recovered from the same patient, carried both the *bla*_OXA-181_ and *armA* genes on a single IncA/C-type plasmid [31].

Three out of the four VIM- and ArmA-producing *P. mirabilis* carried the two corresponding genes on IncA/C- and IncR-type plasmids, respectively. The remaining *P. mirabilis* isolate carried both *armA* and *bla* VIM-1 genes IncA/C plasmid [32].

### 3.6. Association between the RMTase RmtF and Carbapenemases

Out of the 25 *rmtF*-positive isolates (24.3%, the second most commonly identified RMTase-encoding gene in our study), all were *K. pneumoniae* that mainly belonged to ST231 (*n* = 21), followed by ST16 (*n* = 2) and ST147 (*n* = 2). Eight out of those 25 RmtF-producing *K. pneumoniae* produced the KPC-2 carbapenemase, the corresponding *bla*_KPC-2_ gene being located on an IncFII-type plasmid [28]. Six isolates produced the OXA-232 carbapenemase, the latter gene being identified on IncFIB-type (*n* = 4), IncA/C-type (*n* = 1), and ColE-type (*n* = 1) plasmids [12]. Two isolates produced the carbapenemase NDM-1, both the *rmtF* and the *bla*_NDM-1_ genes being co-located on the same plasmid in both cases, namely an IncR-type plasmid in one isolate and a PBRT-untypeable plasmid in the other [33]. One out of the RmtF-producing *K. pneumoniae* isolates co-produced the two carbapenemases OXA-181 and NDM-5, the *rmtF* and the *bla*_NDM-5_ genes being co-located on a single IncR-type plasmid in one isolate.

Noteworthy, seven carbapenem-resistant isolates did not produce any carbapenemase, and all harbored the *rmtF* on an IncFIB plasmid.

### 3.7. Association between the RMTase RmtB and Carbapenemases

Seven *E. coli* and seven *K. pneumoniae* were positive for *rmtB* (13.6%), those isolates belonging to a variety of various ST. All the *E. coli* except one co-produced RmtB and NDM-5 [34]. Four carried IncFIA-*rmtB* and IncA/C-*bla*_NDM-5_ plasmids [35,36]. For two isolates, the *rmtB* and *bla*_NDM-5_ genes were co-located on a unique plasmid being, respectively, IncY and IncFII [27,28]. A single carbapenem-resistant RmtB-positive *E. coli* isolate did not produce any carbapenemase and carried the *rmtB* gene on an IncY plasmid.

Among the seven RmtB-producing *K. pneumoniae*, three possessed a *bla*_OXA-48-like_ gene (two *bla*_OXA-181_ and a single *bla*_OXA-48_) co-located on an IncA/C plasmid [37]. The last four isolates co-produced two carbapenemases, namely an OXA-48-like enzyme (two OXA-48, one OXA-181, and one OXA-232) with an NDM-like enzyme (two NDM-1 and two NDM-5). A single isolate co-produced OXA-181, NDM-5, and RmtB with all three genes being co-located on a same IncFII plasmid. A single *K. pneumoniae* isolate possessed an IncFII-*bla*_OXA-48_-RmtB plasmid and an IncA/C-*bla*_NDM-1_ plasmid. Another *K. pneumoniae* isolate carried both the *rmtB* and *bla*_NDM-5_ genes on the same PBRT-untypeable plasmid, together with a *bla*_OXA-232_ gene on a ColE-type plasmid. Finally, one *K. pneumoniae* isolate possessed an IncA/C-*rmtB* plasmid, together with PBRT-untypeable *bla*_NDM-1_- and *bla*_OXA-48_-carrying plasmids.

### 3.8. Association between the RMTase RmtC and Carbapenemases

Five isolates (4.9%) of our collection, namely one *E. cloacae*, one *E. xiangfangensis*, one *E. coli*, one *M. morganii*, and one *C. freundii*, produced RmtC. In all those isolates, the *bla*_NDM-1_ and *rmtC* genes were co-located on an IncA/C-type plasmid [38]. The *E. xiangfangensis* isolate additionally possessed an IncL/M-*bla*_OXA-48_ plasmid.

**Table 3:** Association of 16S RMTases, carbapenemases, sequence types, and plasmid incompatibility groups among pan-aminoglycoside- and carbapenem-resistant *K. pneumoniae*.

*K. pneumoniae* (n = 75)		Sequence Type	Inc-Type Methylase	Inc-Type Carbapenemase	Co-localisation	References
ArmA (n = 34)						
	KPC-2 (n = 5)	ST 101 (n = 4)	FII	FII	Yes	[29]
		ST 16 (n = 1)	FII	FII	Yes	[29]
	KPC-3 (n = 4)	ST 101 (n = 4)	FII	X3 (n = 1)	No	
				L/M (n = 1)	No	
				A/C (n = 1)	No	
				R (n = 1)	No	
	NDM-1 (n = 12)	ST 11 (n = 1)	A/C	A/C	Yes	[26]
		ST 16 (n = 2)	A/C	A/C	Yes	[26]
			FII	FII	Yes	[28]
		ST 147 (n = 5)	A/C	A/C	Yes	[26]
		ST 247 (n = 5)	A/C	A/C	Yes	[26]
		ST 395 (n = 1)	A/C	A/C	Yes	[26]
		ST 405 (n = 1)	A/C	A/C	Yes	[26]
		ST 437 (n = 1)	A/C	A/C	Yes	[26]
	Carbapenemase-negative (n = 4)	ST 14 (n = 1)	-	Untypeable	-	
		ST 15 (n = 1)	-	FIB	-	
		ST 101 (n = 1)	-	R	-	
		ST 340 (n = 1)	-	Untypeable	-	
	OXA-48 (n = 8)	ST 231 (n = 1)	Untypeable	R	No	
		ST 395 (n = 2)	L/M	A/C	No	
		ST 437 (n = 3)	FIB	A/C	No	
		ST 944 (n = 2)	L/M	A/C	No	
	OXA-181 (n = 1)	ST 35 (n = 1)	A/C	A/C	Yes	[37]
RmtF (n = 25)						
	KPC-2 (n = 8)	ST 231 (n = 8)	FII	B/O	No	This study
	NDM-1 (n = 2)	ST 11	R (n = 1)	R	Yes	[33]
			Untypeable (n = 1)	Untypeable	No	
	Carbapenemase-negative (n = 7)	ST 231 (n = 5)	FIB	-	-	[12]
		ST 16 (n = 2)	FIB	-	-	
	OXA-48 (n = 1)	ST 147	FIB	L/M	No	
	OXA-181 + NDM-5 (n = 1)	ST 147	R	R (NDM-5), untypeable (OXA-181)	Yes (NDM-5 +RmtF)	[33]
	OXA-232 (n = 6)	ST 231	FIB (n = 5)	colKp3	No	[12]
			Untypeable (n = 1)	A/C	No	
RmtB (n = 7)						
	OXA-48 (n = 1)	ST 101	A/C	A/C	Yes	[37]
	OXA-48 + NDM-1 (n = 2)	ST 11	A/C	Untypeable	No	
		ST 101	FII	FII (OXA-48), untypeable (NDM-1)	Yes (OXA-48 + RmtB)	[28]
	OXA-181 (n = 2)	ST 16	FII	FII	Yes	[37]
		ST 147	A/C	A/C	Yes	[37]
	OXA-181 + NDM-5 (n = 1)	ST 16	FII	FII (OXA-181 + NDM-5)	Yes	[37]
	OXA-232 + NDM-5 (n = 1)	ST 231	A/C	Untypeable (OXA-232, A/C (NDM-5)	Yes	[37]
RmtG (n = 1)	KPC-2	ST 11	B/O	FIB	No	[12]
RmtB + RmtF (n = 1)						
	OXA-181 + NDM-5	ST 16	FII (RmtB), untypeable (RmtF)	FII (OXA-181), untypeable (NDM-5)	Yes (OXA-181+RmtB)	[37]
Non-RMTases (n = 4)						
	KPC-2 (n = 2)	ST 11	-	FII	-	
		ST 101	-	FII	-	
	KPC-3 (n = 1)	ST 1519	-	FII	-	
	OXA-48 (n = 1)	ST 147	-	L/M	-	

**Table 4:** Association of 16S RMTases, carbapenemases, sequence types, and plasmid incompatibility groups among pan-aminoglycoside- and carbapenem-resistant *E. coli*.

*E. coli* (n = 12)		Sequence Type	Inc-Type Methylase	Inc-Type Carbapenemase	Co-localization	References
ArmA (n = 4)						
	NDM-1 (n = 2)	ST 8714	A/C	A/C	Yes	[26]
	NDM-5 (n = 1)	ST 90	A/C	A/C	Yes	[34]
	OXA-181 (n = 1)	ST 167	A/C	A/C	Yes	[37]
RmtB (n = 7)						
	NDM-5 (n = 6)	ST 167	Y	Y	Yes	[34]
		ST 354 (n = 3)	FIA	A/C	No	[35]
		ST 405	FIA	A/C	No	[35]
		ST 2851	FIA	A/C	No	[35]
	Carbapenemase-negative (n = 1)	ST 6823	Y	-	-	
RmtC (n = 1)						
	NDM-1	ST 2520	A/C	A/C	Yes	[38]

### 3.9. Association between the RMTase RmtG and Carbapenemases

A single isolate only produced RmtG, being a KPC-2-producing *K.* pneumoniae. The corresponding genes were located on an IncFIB-type and an IncB/O plasmid [10].

### 3.10. Association between the RMTase RmtB, RmtF and Carbapenemases

One single isolate co-produced two carbapenemases, OXA-181 and NDM-5, and two RMTases, RmtF and RmtB. The *rmtF* and *rmtB* genes were located on PBRT-untypeable plasmids and the *bla*_OXA-181_ and *rmtB* genes were co-located on an IncFII-type plasmid, whereas both *bla*_NDM-5_ and *rmtF* were co-located on an IncR-type plasmid.

### 3.11. Association between non-RMTase-Related but Pan-Aminoglycoside-Resistant and Carbapenemases

Four carbapenem-resistant *K. pneumoniae* isolates (3.9%) were pan-resistant to aminoglycosides, but did not produce any RMTase. They respectively belonged to ST11, ST101, ST 147, and ST 1519. Two isolates produced KPC-2, one produced KPC-3, with the *bla*_KPC_ genes being located on IncFII-type plasmids, while a single isolate produced OXA-48, of which the corresponding gene was located on the epidemic IncL-type plasmid.

## 4. Discussion

This retrospective molecular epidemiological study identified a high rate of pan-aminoglycoside among carbapenem-resistant *Enterobacterales* isolates recovered at the Swiss National Reference Center for Emerging Antibiotic Resistance (NARA) between 2017 and 2020. This rate of 10.4% (103/991) (which is quite high) explains why virtually all ß-lactams and all aminoglycosides may not be efficient for treating infections associated with those strains. Plazomicin, a novel aminoglycoside (not marketed yet), is not submitted to modification by aminoglycoside-modifying enzymes. However, the modification of its same target, the 16SrRNA by those RMTases, will make it nonefficient as well. A significant rate (4.4%, 44/991) of carbapenem-resistant and aminoglycoside-resistant enterobacterial isolates co-harbored the carbapenemase and the RMTase genes on the same plasmid. This partially explains the co-occurrence of those threatening and clinically relevant resistance determinants in many clinical isolates. Interestingly, the RMTase genes were never carried on the IncL-type plasmid pOXA-48, being by far the most common plasmid carrying the *bla*_OXA-48_ gene worldwide. This is reassuring, meaning that those RTMase genes may not use the same transmissible vector as that of this carbapenemase gene spreading at very high frequency among enterobacterial species.

In our collection, the proportion of RMTases producers was twice higher among NDM producers than among those producing KPC- or OXA-48-like carbapenemases.

The association of NDM and ArmA was the most commonly observed, emphasized in the majority (22.3%) of the isolates. Such an association was overall not related to the spread of some successful clonal strains, with the corresponding isolates being of different species but with a major plasmid scaffold, pNDM-1_Dok01. This plasmid of the IncA/C incompatibility group plasmid type extensively disseminated among *Enterobacterales* [26]. It is worth highlighting the limited therapeutic options to treat severe infections mediated by carbapenem- and pan-aminoglycoside-resistant *Enterobacterales*, therefore being considered as multidrug-resistant.

Here, we showed that the annual rate of RMTases among carbapenemase-producing *Enterobacterales* showed an increasing trend, from 7.5%, 10.7%, and 11.2% to 13% from 2017 to 2020. These results are in the range of those described among *K. pneumoniae* in Greece, but still less than the prevalence of 42.5%, 56.9%, and 93.4% reported in India, the UK, and China, respectively [8,20,39,40].

This epidemiological study showing the frequent association of RMTases and carbapenemases among carbapenem-resistant enterobacterial isolates in Switzerland further highlights the necessity to implement adapted surveillance strategies and infection control measures, in order to prevent the occurrence of outbreaks caused by such isolates. This control can be facilitated by the implementation of screening strategies at hospital admission, using adapted selective media. This is easily feasible considering that corresponding tools are available nowadays, not only for carbapenem-resistant isolates with a series of selective media for carbapenem-resistant *Enterobacterales* showing high performances, but also for pan-aminoglycoside-resistant *Enterobacterales* with the use of the recently developed SuperAminoglycoside selective medium showing high specificity and sensitivity [41,42,43].

Such a frequent association between carbapenemase-producing and RMTase-producing *Enterobacterales* was already reported in different parts of the world [5,6,7,8,9,10,11,12,13,21,27,28,29,30,31,32,33,34,35,36,37]. The origin of those isolates being identified in Switzerland could be autochthonous theoretically; nevertheless, clinical information (only partially obtained and therefore not presented in that study) showed that many of them corresponded to imported cases, and particularly from Eastern European countries, from Italy, and from the Indian subcontinent.

It should be noted that a phenotype of pan-resistance to aminoglycosides without production of RMTases was very rarely observed (only identified in four isolates) and likely corresponded to the co-production of several aminoglycosides-modifying enzymes. This basically underlines the major role played by RMTase in pan-aminoglycoside resistance. Consequently, acquisition and dissemination of RMTases should be better monitored, considering the crucial role aminoglycosides actually play in clinical practice on a daily basis. It is worth mentioning that ca. 1% of the enterobacterial strains received in this Swiss National Reference Center are resistant to colistin, aminoglycosides, and β-lactams, paving the way to impossible-to-treat infections. Finally, this study underlines that it is now time to consider not only carbapenem resistance but also pan-aminoglycoside resistance among the most worrying and emerging threats in *Enterobacterales*, taking into account the clinical importance of this antibiotic family in the management of infectious diseases.

## Figures and Tables

**Table 1:** Total number of 16S RMTases detected in pan-aminoglycoside and carbapenem-resistant *enterobacterial* species between 2017 and 2020 from the NARA.

Species	Negative	ArmA	RmtB	RmtC	RmtF	RmtG	RmtB + RmtF	Total
*Citrobacter freundii*	0	0	0	1	0	0	0	1
*Enterobacter cloacae*	0	2	0	1	0	0	0	3
*Enterobacter hormaechi*	0	1	0	0	0	0	0	1
*Enterobacter xianfangensis*	0	1	0	1	0	0	0	2
*Escherichia coli*	0	4	7	1	0	0	0	12
*Klebsiella pneumoniae*	4	34	7	0	25	1	1	72
*Morganella morganii*	0	0	0	1	0	0	0	1
*Proteus mirabilis*	0	6	0	0	0	0	0	6
*Providencia rettgeri*	0	2	0	0	0	0	0	2
*Providencia stuartii*	0	3	0	0	0	0	0	3
Total	4	53	14	5	25	1	1	103

**Table 2:** Total number of carbapenemase and 16S RMTases detected in pan-aminoglycoside and carbapenem-resistant *Enterobacteriaceae* between 2017 and 2020 from the NARA.

16S RMTases	Carbapenemase Negative	NDM-like	OXA-48-like	NDM- + OXA-48-like	KPC-like	VIM-like	Total
Negative	0	0	1	0	3	0	4
ArmA	5	23	12	0	9	4	53
RmtB	1	6	3	4	0	0	14
RmtC	0	4	0	1	0	0	5
RmtF	7	2	7	1	8	0	25
RmtG	0	0	0	0	1	0	1
RmtB + RmtF	0	0	0	1	0	0	1
Total	13	35	23	7	21	4	103

## Data Availability

The data presented in this study are all available in the main text.
